# The New York Institute of Stomatology

**Published:** 1902-12

**Authors:** 

**Affiliations:** The New York Institute of Stomatology


### Reports of Society Meetings.

### THE NEW YORK INSTITUTE OF STOMATOLOGY.

A meeting of the Institute was held at the office of Dr. C. D. Cook, No. 162 Remsen Street, Brooklyn, N. Y., on Tuesday evening, June 3, 1902, the President, Dr. Howe, in the chair.

The minutes of the last meeting were read and approved.

### COMMUNICATIONS ON THEORY AND PRACTICE.

*Dr. F. Milton Smith.*—A practical point occurs to me, and the satisfaction I have had in its use prompts me to speak of it. Our president, Dr. Howe, called my attention to it first. It is the use of the precipitate of silver for absorbing the excess of mercury from amalgam fillings. Formerly I used the plastic forms of gold, but the gold is not nearly so efficacious. I am in the habit of getting it from Eimer & Amend, Third Avenue and Eighteenth Street, New York. It is a powder, and costs one dollar and twenty-five cents an ounce. Dr. Howe tells me that his method of using it is to make his filling a little full and then cover it with the precipitate of silver; the precipitate and surplus mercury will come off in a scale, leaving the filling clean.

*Dr. Shaw.*—May I ask what advantage the precipitate has over a portion of amalgam squeezed very dry?

*Dr. Smith.*—I invariably do squeeze all the mercury possible out of the amalgam, but even then there will be an excess which the precipitate of silver takes up very nicely.

*Dr. A. H. Brockway.*—I have always thought it of very great advantage to mallet in my amalgam fillings under a pellet of bibulous paper; in this way if there is any excess of mercury it is brought to the surface, where it can be readily wiped off or absorbed. I should suppose that this precipitate of silver would be of advantage in effecting this absorption where necessary. I have sometimes used pellets of gold and sometimes tin-foil. I think the malleting is a decided advantage.

Dr. W. M. Whitlock then read his paper entitled "Importance of Details."

(For Dr. Whitlock's paper, see page 893.)

*The President.*—We are very much indebted to Dr. Whitlock for the painstaking care with which he has presented this subject. Discussion of the subject is now in order.

DISCUSSION.

*Dr. Chas. O. Kimball.*—I think we should thank Dr. Whitlock for the phraseology with which he has introduced this subject. The question of details in our work can never be brought too closely before us. With our different ways of working we sometimes fall into habits that are not the best, and so a careful presentation of any man's carefully thought out method of doing small things I have found, in my own case, to be of the greatest assistance.

There is one thing Dr. Whitlock mentioned that I would like to call special attention to, and that is the measurement of roots and the marking of broaches. It has been my habit for a good many years to have in my pocket a little scale marked to thirty-seconds of an inch, and in opening roots I measure them in all cases. I mark the instrument used for this purpose with a bead of shellac about three-quarters of an inch from the end. This is done by warming the instrument and touching with a stick of heated shellac. Formerly I used to mark the instrument with a file nick, but the objection to this is that it makes a weak point in the instrument, and a bit of rubber dam, which is sometimes recommended, is liable to slide; but with a bead of shellac on a probe you can go into a canal to the point desired and there stop with the greatest certainty. For instance, in a tooth where there is a marked opening at the apex, with a fine broach with a little hook on the end the exact length of the canal can be ascertained; then by marking a plugging instrument in this way, you can feel to a certainty that your filling-material goes to and no farther than the apex.

As for sharpening pluggers in the manner described, by passing them through a little piece of wood and sharpening on the oil-stone, I found years ago that a little piece of hickory well dried has a loose porosity through which you can pass the end of a broach.

The piece of wood needs to be about one-eighth of an inch long, cut squarely across the grain. An instrument so sharpened, if examined with the glass, will be found to have a perfectly square end with sharp edges on all sides so that they will catch the most minute fibres of silk and carry them up into the root, while an instrument sharpened in the usual manner by holding between the fingers will show the sides rounded so that it will slip through the material that is being carried into the root.

These two points, it seems to me, will bear emphasizing very strongly, using always marked instruments when examining and filling canals and sharpening plugging instruments with clean square surfaces in the manner described.

*Dr. S. E. Davenport.*—We are indebted to Dr. Whitlock for his careful presentation of this subject, and particularly for the accuracy of the system he has devised. Our Institute is certainly a body before which to bring the description of a carefully systematized method, for I believe we appreciate all such and frown upon the reverse. A point which has impressed my mind, because of some recent events, was the suggestion made by Dr. Whitlock that we do not attempt to open into the roots of a molar from a posterior approximal or a buccal cavity. This last week I have seen in the office of my associate a most lamentable result of an attempt on the part of a dentist in another city to open into the roots of a third molar through a buccal cavity.

*The President.*—I believe there is material for an evening's discussion upon this subject, but as we are not prepared to go into it to-night we will listen to the next paper on the programme, by Dr. Fuller.

### A BRIEF ADDRESS.

BY DR. D. A. FULLER.

Mr. President and Gentlemen of the Institute,—Just a few words on the use of sulphate of quinine in the treatment of periodontitis, especially when patients give history of malarial poisoning. In making a few notes on a rather interesting case that came under my care during last July I am aware that there is something of a prejudice existing among the medical and dental profession regarding the use of quinine. For my own part I am very much in favor of the drug and believe the good effects far outbalance the deleterious.

The patient in question was Mrs. F., the wife of a college professor. She came to my office on Monday, July 8, with all the symptoms of an incipient alveolar abscess of the left inferior second bicuspid.

The local dentist of the town where the lady came from had removed the filling from the tooth, and I found the pulp-canal open at the time. She described the pain as having been intense, and had slept but little during the previous forty-eight hours. I examined the tooth and gave her but little encouragement of preserving it (except in alcohol), especially as she wished to start on a Canadian trip the latter part of the following week. However, I told her that we could at least give the tooth a trial and extract as a last resort. She informed me that she would remain a few days in Brooklyn, if necessary, for treatment.

I first syringed the tooth repeatedly with hot water and then placed a piece of cotton very loosely in the cavity to keep out any foreign matter. I also ground the cusp on the opposing tooth so that the two teeth did not meet. In the mean time I had learned that the patient was subject to frequent attacks of malaria, and I decided to give the quinine a thorough trial during that day. It was then about ten o'clock. I instructed the patient to take eight grains of quinine at once; in fact, I sent out for it and saw that it was done. I then advised her to continue it, in two-grain doses, during the day at intervals of about an hour; and with these instructions she left the office.

She called me up on the 'phone about five o'clock that evening and said that the intense pain had about subsided. The next day she 'phoned me again and reported that she had slept very well, indeed, during the night, and would not visit me that day unless I thought it necessary. I instructed her to keep on with the quinine, taking the doses rather less frequently than the day before, with a limited diet.

She came back the next morning, which was Wednesday, and, as an Englishman would say, was in "a very fit condition." The tooth had receded almost to its normal position, and had tightened very perceptibly in the socket, showing that the inflammatory condition was greatly relieved. I will not elaborate on the future treatment except possibly the latter part.

I sent her to her country home the latter part of that week in a very comfortable condition. I placed an antiseptic dressing in the canal, with cotton and sandarach over this. The lady returned to

Brooklyn the first part of the following week, and I placed a temporary filling in the root and filled the crown with soft gutta-percha, easily removable by the patient in case of recurrence of the lesion.

Mrs. F. went to Canada for two or three weeks and returned to Brooklyn on October 8, having experienced no difficulty whatever, and so far as I know, up to the present time, no unfavorable symptoms have developed.

A rather curious incident in connection with this case was my discovery that the tooth directly in front of this (the first bicuspid) contained a devitalized pulp. I treated and filled this tooth permanently during the first week of Mrs. F.'s visit to Brooklyn.

In conclusion, then, I will say that for the past six or seven years I have prescribed quinine in a large number of cases, and have almost invariably found that the patient has been relieved and a considerable number have been very greatly benefited.

Where the malarial symptoms are prominent in an asthenic person is where, in my opinion, the use of quinine is indicated.

DISCUSSION.

*Dr. Davenport.*—I would like to ask Dr. Fuller whether in this case he did any less for the tooth in the way of treatment than he would had he not prescribed quinine?

*Dr. Fuller.*—No. I gave the tooth the same treatment that I would have given it in all ordinary cases, but I find the quinine helps me out in these cases many times. I had a case the other day, to diverge a little, in a patient who had suffered a great deal in an attempt to devitalize a tooth. I found, upon questioning her, that she was subject to malarial attacks, and I put her under the quinine treatment. I did not apply the arsenic that night, but after washing out with hot water I applied a sedative to the pulp. The next day, while all the pain had not subsided, she was greatly relieved, so much so that I applied arsenic by drilling through the crown of the tooth, putting in a minute bit of arsenic and morphia, and something in the large cavity to quiet the tooth. I also kept her under the quinine treatment. That was three or four days ago. She came to the office yesterday, when I gave her a half-hour sitting and removed the pulp.

*Dr. Brockway.*—I would like to ask Dr. Fuller if he employs any other means than simply washing out the tooth with hot water. It seems to me that it would have been well to have gone into the root and cleansed it out mechanically as much as possible.

*Dr. Fuller.*—I think I stated in the case I mentioned that the tooth had already been treated. The fact is, it had been pretty nearly treated to death, and was so loose that I could have taken it out with my fingers.

*Dr. Brockway.*—I asked because possibly Dr. Fuller may have thought he could not work on the tooth because of its extreme soreness. In these cases where a tooth is so tender that it is practically impossible to work on it I have had excellent success by applying a Perry separator, thus holding it firm. I remember one case of a lady who came to me with a dying pulp in a third molar. The tooth was so tender that she could not close her mouth without causing great pain. By applying a separator I was able to open and thoroughly disinfect the tooth in an entirely satisfactory manner. I wish to commend the use of hot water. I place great reliance in it.

*Dr. Davenport.*—The reason I asked Dr. Fuller the question I did is that we are apt to attribute recovery to the last medicine that has been taken. One part of the treatment he administered was enough in itself to aid in the recovery of the tooth, and that was what Dr. Codman, of Boston, used to call the "grindstone cure." If the tooth itself is too tender to grind, then do as Dr. Fuller did, grind the occluding tooth. That alone is often sufficient to cause the tooth to get well.

*Dr. F. Milton Smith.*—I have never had any experience in the use of quinine for the relief of such conditions as Dr. Fuller has mentioned, but I have had many cases where liberal doses of quinine relieved people suffering, some of them for weeks, with a form of neuralgia, the cause of which seemed almost impossible to trace. Because of this I have confidence in liberal doses of quinine in obscure cases of neuralgia which manifests itself in the nerves of the jaws. I have seen a number of cases where liberal doses have, within forty-eight hours, driven away and kept away the neuralgia, the treatment being continued for some days. Sometimes the pain has not appeared again for many weeks, when it has been necessary to repeat the treatment.

If I may digress a little I would like to mention the very great help I have had in the use of a string tied around very sore teeth where it is necessary to open them for relief. During the absence of Dr. Allen this spring one of his little patients, about ten years of age, who had broken off a tooth by a fall, and in which the pulp had died, fell into my hands. When the little fellow came in the tooth was so sore he could hardly bear the touch of his tongue to it. I tied a silk ligature around the tooth and then told the little fellow that if he would carefully draw on that till he got it real tight I would see what I could do. It was a revelation to me. I drilled into the tooth and took the pulp out as prettily as could be and without an expression of pain from the little fellow.

Dr. J. Morgan Howe read a paper entitled "Notes on Methods of preventing Decay."

(For Dr. Howe's paper, see page 888.)

DISCUSSION.

*Dr. Kimball.*—I wish to thank Dr. Howe for adding his word in connection with the conservation of the teeth, for I have felt very strongly that while we may and should remove disorganized tissue, or that which is only partially disorganized, cutting away sound tissue for a possible future good while we have other means at our command which may produce the same result has seemed to me hardly justified. So long as we find, in cases of teeth markedly liable to decay, that when we can induce the patient to care for them properly we can check decay, even in such a mouth, it does not seem to me quite fair to the teeth to cut away sound tissue for the mere purpose of preventing decay. As for the means made use of for doing that it is not necessary for me to speak. It is by, I might say, "hourly care," or, rather, meal-time care, cleaning the teeth whenever they are soiled, as invariably as you wash your hands whenever they are soiled, and in such a way that the teeth are made clean. You and I—all of us—have seen the result of such care in our practice. How it may be in some cases where patients will not give the proper care I am not prepared to say.

*Dr. Smith.*—One thought that Dr. Howe brought out in his paper, it seems to me, we ought to always have in mind in filling approximal cavities, and that is that the joint between the metal and the tooth should not come in contact with the adjoining tooth. It seems to me this is the secret of success in filling teeth that are decayed on approximal surfaces. This can be done in very many cases, if the teeth are sufficiently separated, by cutting away comparatively little tooth-structure. I have in my own practice many cases that have been filled in this way with comparatively small gold fillings. The fillings are so contoured out that when the teeth come together nothing but gold touches. It would seem to me folly to cut away sufficient to hide all the margins under the gum or bring them out to the surface.

*Dr. Kimball.*—May I ask a question? I recently had occasion to wedge apart a couple of teeth for a young lady twenty-two years of age, who, to my knowledge, has been extremely careful in cleansing her teeth, keeping them in very good condition. There was trouble between the right lower first and second molars, and I wedged them apart pretty thoroughly. I found the point where the teeth came in contact to be sound, but there was a spot of decay on either side of this point, just inside and just outside, two marked cavities in each tooth. The section of tooth between them was sound, just a little discolored. In treating them I cut through, making one cavity, and it was pretty hard work. Now, why did they decay where there was no contact, and why did they not decay where there was contact; and if I had had a gold filling at the point of contact, would not decay have started at the edges of the gold?

*Dr. Davenport.*—It seems to me that Dr. Howe's paper is extremely original. He calls to our mind that, having cut away decayed and softened tissue, we have reached a point where the influences that combine to disintegrate the tooth-structure do not exist, and, as this portion of the tooth has already resisted such influences, asks why it should be removed. This is a point we may well ponder over, for there is a great deal in it. Another point that attracted my attention particularly was the reference to Dr. Arthur's suggestion that after his method had been practised great care be taken of the teeth. Dr. Howe evidently believes that if the increased care be given to the teeth it would be unnecessary to cut away the tooth-structure that Dr. Arthur's disciples remove. We have about arrived at the point of believing that clean tooth-substance does not decay, and if we can by any means increase the care of our patients, it seems to me we will be better able to conserve the tooth-substance.

*Dr. Brockway.*—The point mentioned in Dr. Howe's paper regarding Dr. Arthur's requiring unusual care in cleansing these surfaces also struck me as being particularly sound. I firmly believe that if great care were to be bestowed upon cleansing the teeth it would not be necessary to cut away tooth-structure nor to fill the teeth at all. I believe decay can be almost wholly prevented by proper cleansing, and I hope you have all read the papers of Dr. Smith upon that subject. I think he has contributed more to the successful practice of dentistry, which means the preservation of the human teeth, than almost any other writer upon the subject. It seems to many patients a great task to come to the dentist as often as Dr. Smith suggests for the purpose of having their teeth cleansed and the gelatinous plaques removed, but in speaking to a well-known clergyman upon the subject one day he remarked, "Why, it seems to me that it is no greater task than people are willing to submit to in going occasionally to have their hair cut or in the case of a man going daily or every other day to be shaved." And when you come to think of it, it does not amount to any more. I do believe that if people gave as much time to the cleansing of their teeth by a competent operator as they do to having their hair cut or being shaved, decay would be almost wholly prevented.

*Dr. C. D. Cook.*—Perhaps if the dentist made his fee in proportion to that of the barber, patients would be willing to come more frequently.

*Dr. Brockway.*—But consider how much more important the teeth are. I believe dentists could be brought to take that stand more generally.

*Dr. Cook.*—Referring to the last remark of Dr. Brockway, that if the dentist would take more pains to instruct his patients how to take care of their teeth they would take better care of themselves, I would say that I think dentists do this very thing far more than the general practitioner instructs his patients as to the care they should take of their health.

*The President.*—Dentists, as a rule, perhaps, do more than other professional men to make their work unnecessary. There is no doubt if they do all possible in that line there will still be a great deal to be done.

*Dr. E. A. Bogue.*—I have been told six or eight times within the last few days, by refined lady patients, that never in their lives, until I told them, had they been told how to cleanse their teeth. I am only anxious that we do not flatter the rest of the brethren to such an extent that they go home and fail to tell their patients what they need to do, when all of us in this room know the telling to be so necessary.

Dr. Kimball spoke of a couple of molar teeth that were not decayed at the point of contact, but were decayed on either side of that point. I believe, in every case where I have seen this condition, I have seen defectively formed teeth. Let me call attention, Mr. President, to the fact that some people are born with club feet and some with bow-legs, and some there are who have malformed teeth. I do not mean necessarily distinctly malformed, but malformed perhaps because of the civilization in which they and their ancestors have lived for several generations. In teeth that are flat-sided the sides are too flat to be cleansed by the silk or brush except at the point of contact. These cavities exist because of the shape of the teeth.

Dr. Howe, nearly at the end of his paper, read a pretty long sentence, and I only got a part of it. As I have it, he said, "The time may come when we may produce such changes in the oral fluids as shall tend towards a diminution of decay." This is right in the line of what Dr. Black brought out three or four years ago when he spoke of innoculation against the decay of the teeth. This seems to be based upon the assumption that the fluids of the mouth have to do with decay of the teeth. I am getting around to the point of thinking that this is chimerical, as the saliva, urine, and every other fluid in the body of a healthy person is sterile. Its tendency is not towards decay or the nourishment of bacterial life. I think that after we look into this question more we shall conclude that the so-called plaques of Williams, the temporary immunity from decay of Black, and the condition that we sometimes find in mouths for one or two years and then see the conditions change altogether, are in all cases due to the fact of uncleanliness. I will instance the case of a child that is born tired and that never becomes vigorous. The teeth never were properly formed, and the child never has had gumption enough to properly cleanse them, while the brother or sister of that same child will be a strong and vigorous person. In the case of the latter child, the very way he masticates his food or takes a drink of water tends to cleanse the teeth, while the other does everything in such a languid way that his teeth are never clean. Decay has come from something on the teeth which ought not to be there.

*Dr. Wheeler.*—Have there ever been any comparisons made to note if there is less recurrence of decay in mouths where extension for prevention is practised than in those cases cared for by careful but less radical practitioners?

Adjourned.

FRED. L. BOGUE, M.D., D.D.S.
*Editor The New York Institute of Stomatology.*

